# Supplementary material for: Simulation of the cement measurement based on the pulse DT neutron generator: A Monte Carlo study
Source: PLoS One. 2021 Jun 14;16(6):e0252078. doi: 10.1371/journal.pone.0252078 (PMC8202931; doi:10.1371/journal.pone.0252078)
Supplement: S1 File — (DOCX) [file pone.0252078.s001.docx]

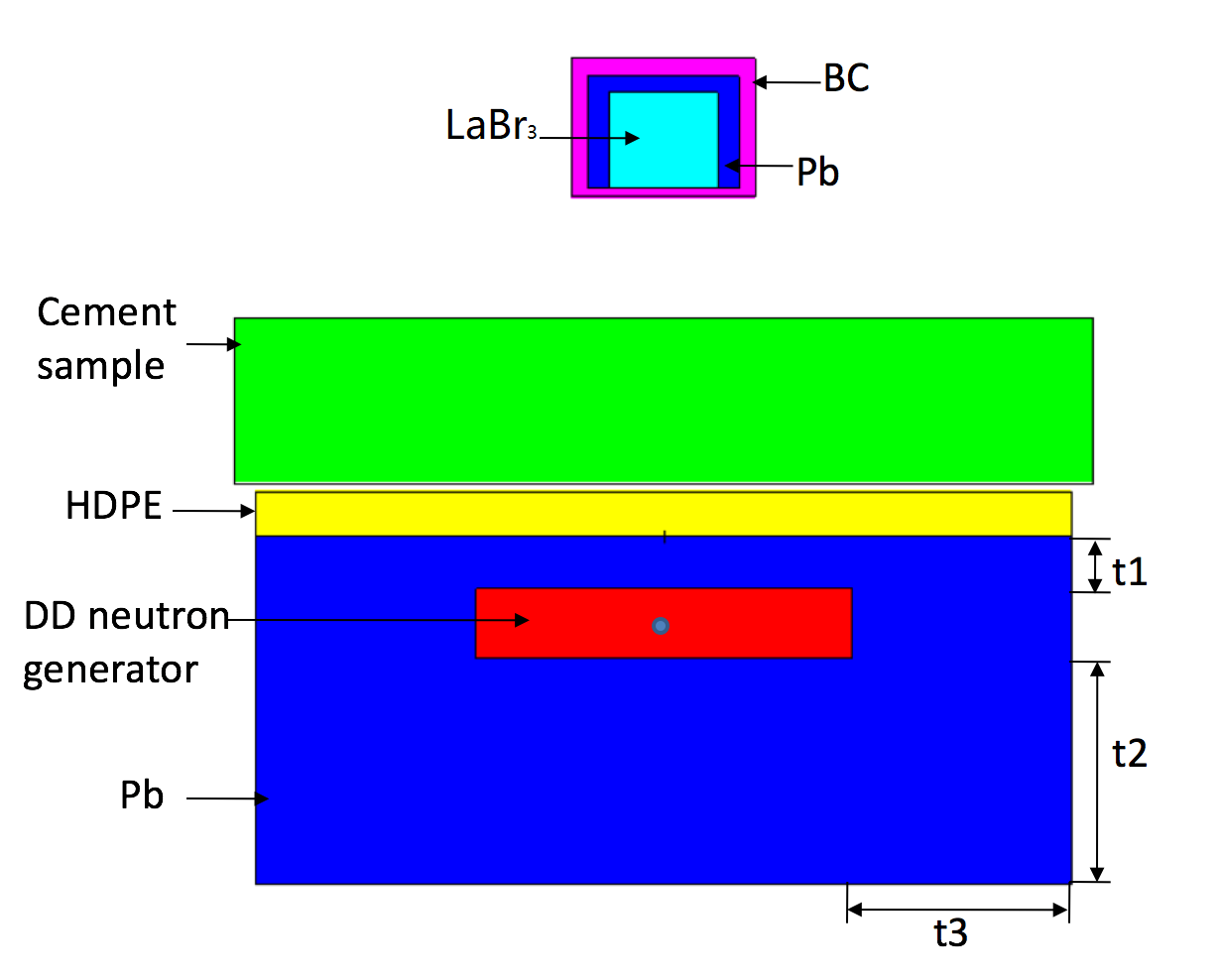


**Fig 1. A schematic of the structure for cement measurement based on DT neutron generator**


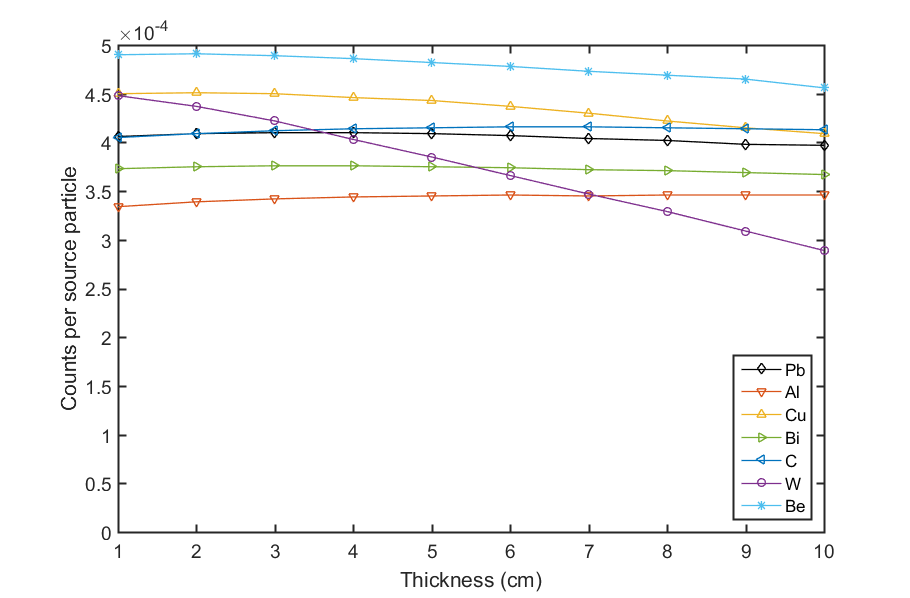


**Fig 2. The neutron flux versus the thicknesses of t1 with different materials.**


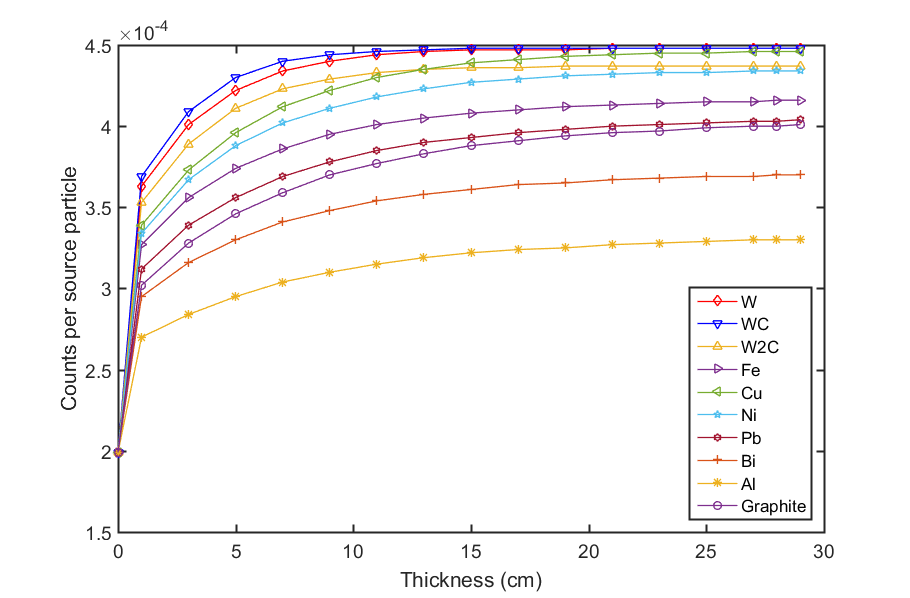


**Fig 3. The neutron flux versus the thicknesses of t2 with different materials.**


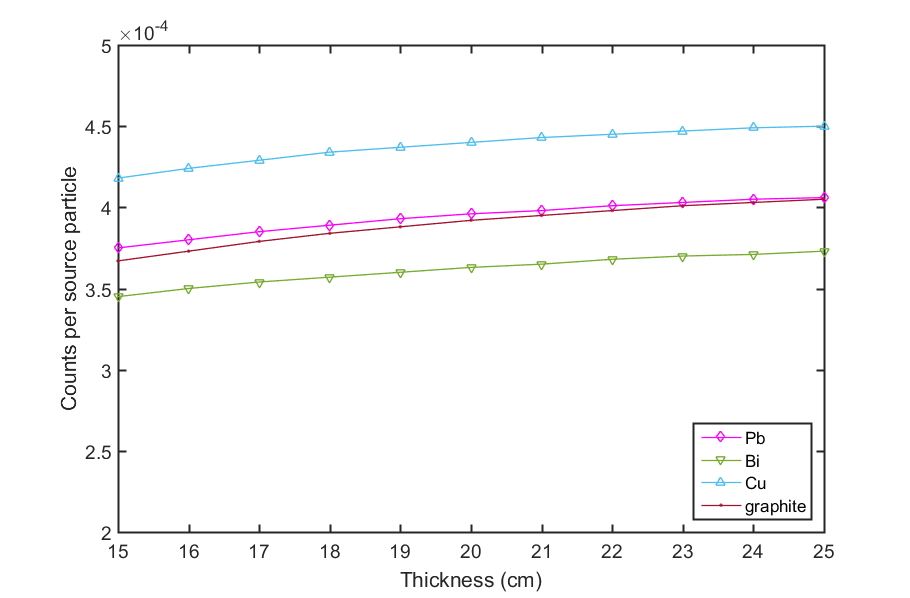


**Fig 4. The neutron flux versus the thicknesses of t3 with different materials.**


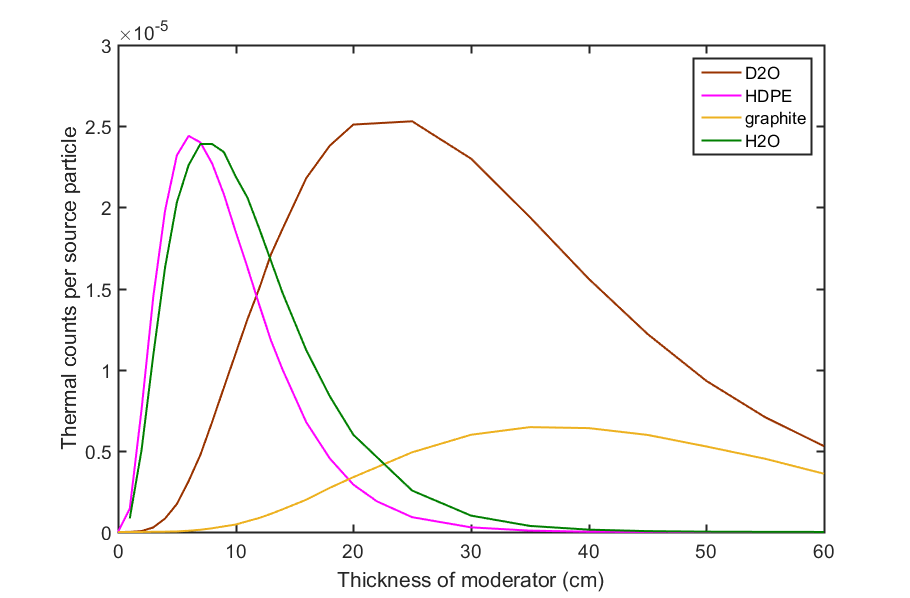


**Fig 5. The thermal neutron flux versus the thicknesses of moderator with different materials.**


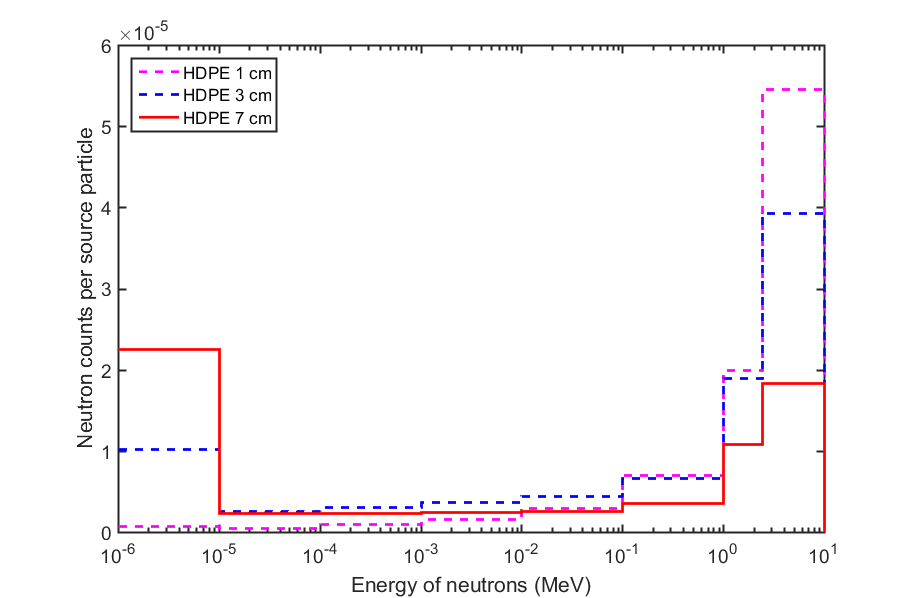


**Fig 6. The energy distributions of neutrons with different thicknesses of HDPE increase.**


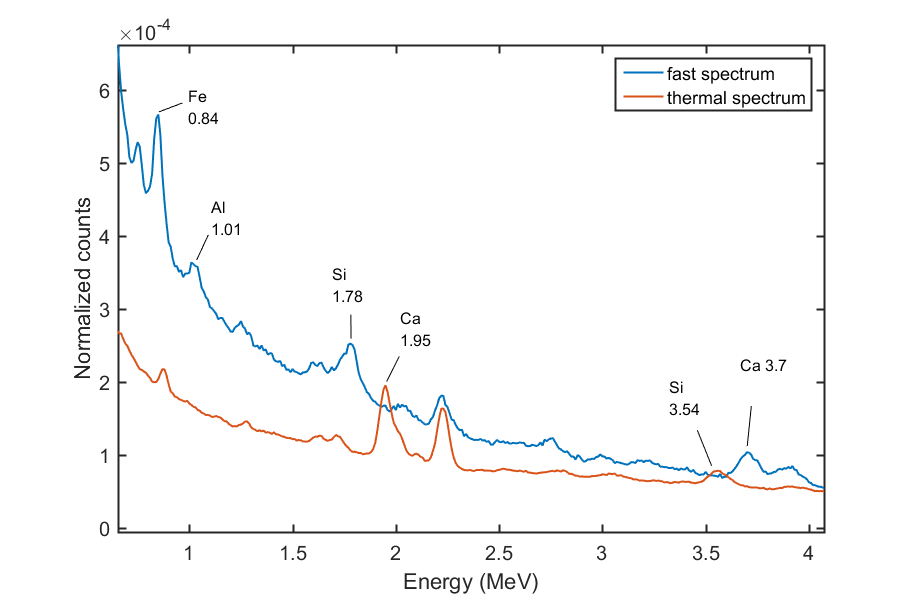


**Fig 7. The gamma-ray spectra at different time intervals at the energy range of 0 to 4 MeV.**


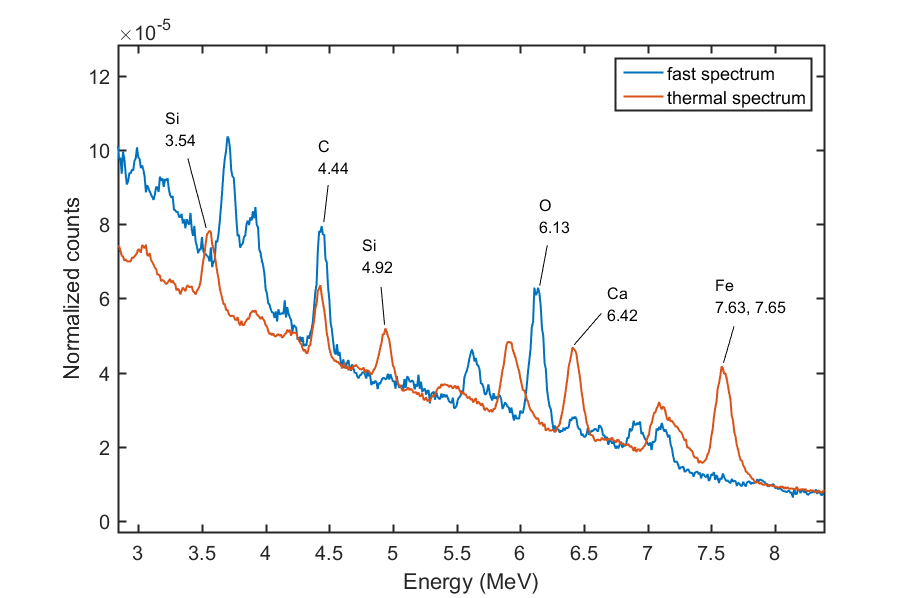


**Fig 8. The gamma-ray spectra at different time intervals at the energy range of 3 to 8 MeV.**


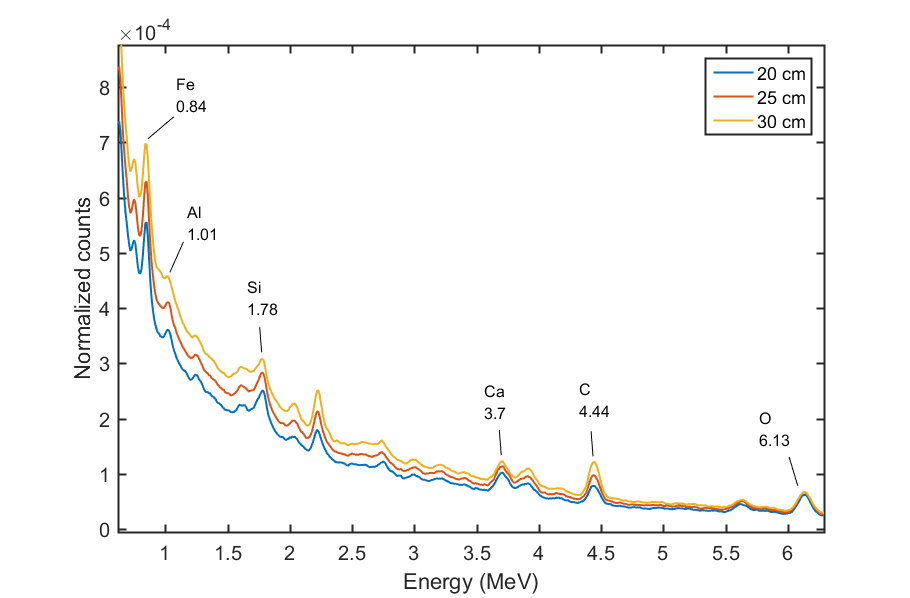


**Fig 9. The fast spectra with different thicknesses.**


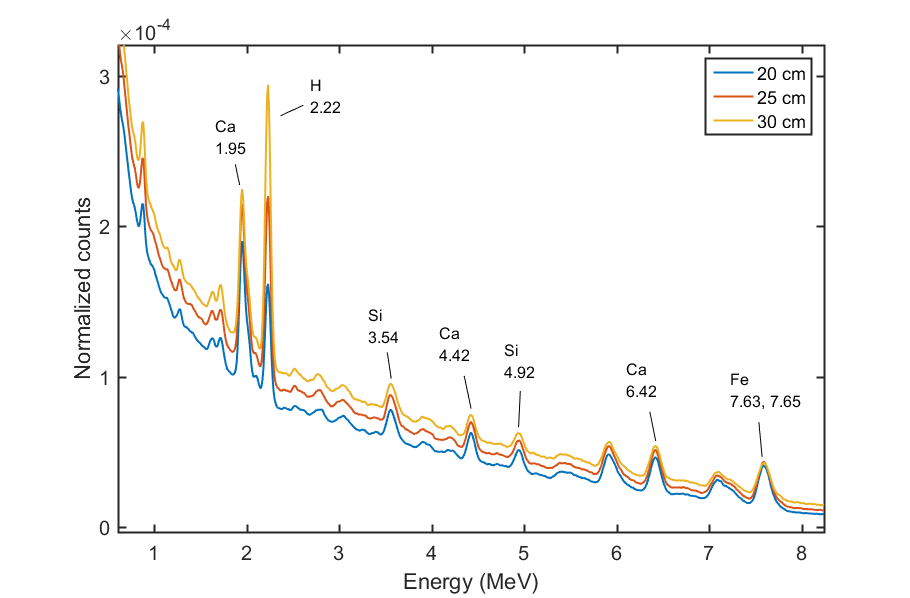


**Fig 10. The thermal spectra with different thicknesses.**


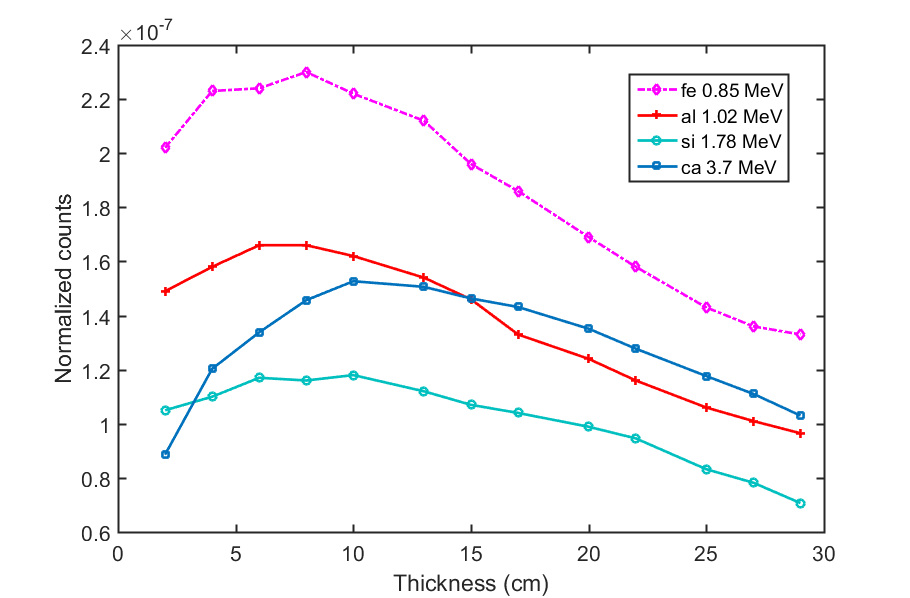


**Fig 11. The characteristic peaks as a function of the thickness of the cement samples.**


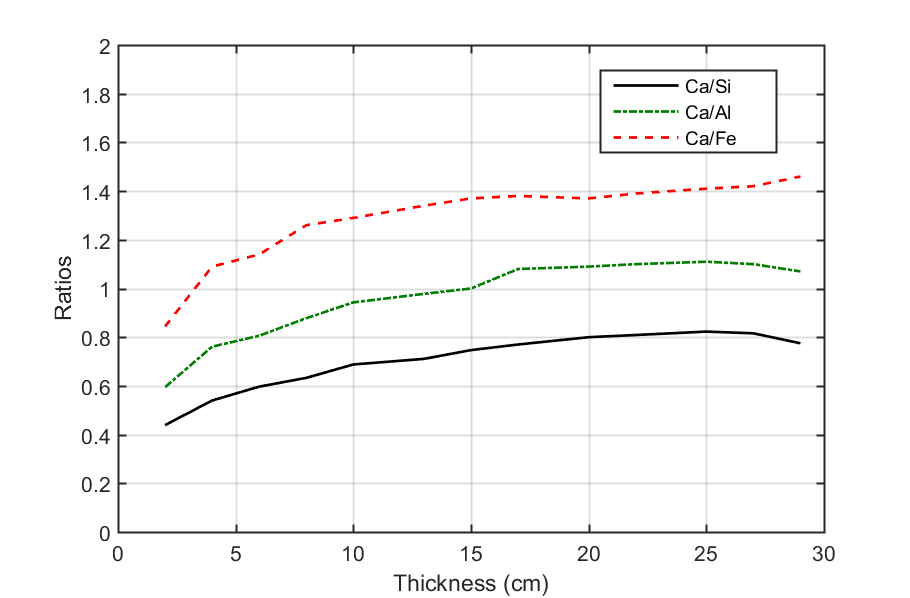


**Fig 12. The ratios of the major elements as a function of the thickness of the cement sample.**
